# Changes of Acid-Base Variables in Dairy Cows with Chronically Implanted Fetal and Maternal Catheters during Late Gestation and Calving

**DOI:** 10.3390/ani12182448

**Published:** 2022-09-16

**Authors:** Ottó Szenci, Gijsbert Cornelis Van Der Weyden, Lea Lénárt, Marcel Antoine Marie Taverne

**Affiliations:** 1 Department of Obstetrics and Food Animal Medicine Clinic, University of Veterinary Medicine Budapest, H-2225 Üllő, Hungary; 2 Department of Farm Animal Health, Foetal and Perinatal Biology, Faculty of Veterinary Medicine, Utrecht University, 3584 CL Utrecht, The Netherlands

**Keywords:** dairy cow, fetal cannulation, maternal cannulation, calving, blood gases, acid-base parameters

## Abstract

**Simple Summary:**

A fetal catheterization is an efficient tool allowing longitudinal in vivo studies on hormonal and metabolic changes, including fetal blood gases and acid-base changes. These surgical techniques made it possible to take blood samples daily under aseptic conditions to determine arterial and/or venous blood samples for acid-base variables (like pH, blood gas tensions: partial pressure of carbon dioxide and partial pressure of oxygen, oxygen saturation, bicarbonate concentration, total carbon dioxide, and base excess). All these examinations may contribute to a better understanding of the physiological changes that occur during calving, which may help reach a significant reduction in losses caused by perinatal mortality, which is still high today.

**Abstract:**

The objective of the present study was to evaluate the changes in maternal and fetal arterial acid-base variables withdrawn from catheterized dams and fetuses during the last days before and during calving. The average gestation length in nine cows with chronically catheterized fetuses was 285 ± 10 (SD) days. The arterial acid-base variables of a catheterized dam and fetus were very stable during late gestation. Four newborn calves showed small differences between prenatal and postnatal pH values (−0.035). At the same time, pCO_2_ values started to increase significantly (*p* = 0.02), indicating a shift towards physiological respiratory acidosis during calving. The partial pressure of oxygen and oxygen saturation values showed some non-significant improvements immediately after birth, while the other acid-base parameters did not differ. The remaining five newborn calves showed a significant decrease in arterial blood pH (*p* < 0.01) and BE (*p* = 0.01), while pCO_2_ tended to be higher (*p* = 0.06), indicating a shift towards physiological respiratory and metabolic acidosis, while the other acid-base parameters hardly differed. It is essential to mention that physiological (*n* = 2) and mild metabolic acidosis (*n* = 2) developed gradually in four newborn calves during the second stage of calving, lasting about ≤ 2 h. In contrast, in the remaining newborn calf the physiological metabolic acidosis developed during the last 3 min of birth because immediately before birth, the BE value was 0.4 mmol/L. After birth, it was −5.4 mmol/L. The results indicate that the acid-base variables may start to move gradually in the direction of expressed respiratory and metabolic acidosis only after appearing the amniotic sac and fetal feet in the vulva during the second stage of labor; therefore, it is essential to complete obstetrical assistance in time.

## 1. Introduction

The prevalence of perinatal mortality (death of a mature fetal calf after at least 260 days of gestation during calving or in the first 24 to 48 h of postnatal life [1,2] is still very high (3.5 to 8%) and contributes to considerable economic losses, especially in Holstein-Friesian dairy farms [3,4]. The perinatal mortality rate in Holstein-Friesian heifers reached a loss of 11 to 13.2% in the last decades [5,6,7,8,9], which nowadays shows a static or declining trend [10]. All of these emphasize the importance of examining the causal factors of perinatal mortality because asphyxia plays a critical role in perinatal mortality (58.3% [11] and 44.7% [12]).

It has been known in human practice for a long time that all fetuses develop more or less severe hypoxia due to the rupture of chorioallantoic and amniotic sacs and uterine contractions consequent metabolic acidosis [13]. Under normoxic conditions, glucose as the primary energy source is reduced to pyruvate via the citric acid cycle to the final products CO_2_ and H_2_O. During oxygen shortage, glucose can only be metabolized anaerobically to pyruvate and then reduced to lactic acid. Anaerobic glycolysis, however, has a significant disadvantage because energy production is reduced, the carbohydrate reserves are rapidly exhausted, and metabolic acidosis develops by the accumulation of acid metabolites (lactic acid). At calving, all fetuses, therefore, suffer from respiratory as well as metabolic acidosis. The degree of respiratory and metabolic acidosis in the perinatal period can be assigned into three groups according to their pH values [14]:

Group 1: blood pH > 7.2–physiological acidosis = slight, combined respiratory and metabolic acidosis.

Group 2: blood pH 7.2–7.0–moderate acidosis = mild to expressed, combined respiratory and metabolic acidosis.

Group 3: blood pH < 7.0–severe acidosis = severe, combined respiratory and metabolic acidosis.

The BE values can also be used to evaluate the degree of acidosis in the perinatal period: physiological acidosis BE <−6.0 mmol/L, moderate acidosis: BE: −6.0 and −12.9 mmol/L, and severe acidosis: ≥−13 mmol/L [15]. The degree of acidosis finally determines whether the fetus lives or dies. Before that, the organism’s regulatory system of chemical buffering in the blood operates to keep the offspring alive. Bicarbonate is the essential buffer. The other ones are hemoglobin, plasma proteins, and phosphate buffers, as reviewed by Szenci [16]. Fetal cannulation may help us understand when respiratory and metabolic acidosis in the fetus to be born can develop to decrease the prevalence of stillbirth on dairy farms.

A significant advance was reached when the method for insertion and maintenance of catheters in umbilical and uterine vessels under chronic conditions was introduced for the sheep and goats by Meschia et al. [17]. Since then, the technique has been widely used for various studies on placental transfer in conscious sheep [18,19] and other large domestic animals such as the cow [20], mare [21,22], and pig [22,23].

The bovine catheter was usually inserted into the umbilical artery and/or vein [20,22,24,25,26] through a placentome selected near the junction of the uterine horns. The anterior tibial artery [24,25,26,27,28,29], saphenous artery [30], or saphenous vein [24,25,26,27,29,30,31,32] were also used to insert a catheter into the caudal fetal artery, aorta and/or fetal caudal vena cava. A fetal medial or metatarsal vein was also tried for cannulation; however, it was often dislodged [20]. The main uterine artery [24,25,26,29,32] and/or vein [22], or a circumflex iliac artery and/or vein [20,27], were usually catheterized on the maternal side of the cow. The maternal jugular vein was also used for the insertion of a catheter [30]. These surgical techniques made it possible to take blood samples daily under aseptic conditions to determine arterial and/or venous blood samples for acid-base variables (like pH, partial pressure of carbon dioxide /pCO_2_/, partial pressure of oxygen /pO_2_/, oxygen saturation /SaO_2_/, base excess /BE/, bicarbonate concentration /HCO_3_^−^/ and total carbon dioxide /TCO_2_/, however, most of the cases only the pH and the blood gas tensions were determined [20,22,30]. Schmidt et al. [26] reported the changes in pH, hemoglobin, pCO_2_, pO_2_, SaO_2_, SO_2_-content, ionized sodium, potassium, chloride, and calcium concentration, and glucose in whole blood in pregnant cows operated under general and local anesthesia while Sangild et al. [29] measured lactate and cortisol concentrations additionally in dams pregnant with in-vivo and AI produced embryos.

Independently of the duration of the surgery, a well-trained surgical team is needed for fetal cannulation to decrease the risk factors during operations [28]. At the same time, Smith et al. [26] also received a good result when pregnant dams for fetal catheterization were performed upon a standing cow in local anesthesia (total duration of the surgery: 2 to 2.5 h) compared with general anesthesia in the dorsal position (total duration of the surgery: 3 to 3.5 h).

Because of the extended duration of obstetrical traction (more than 2 min) [33] or in case of the delayed second stage of labor in primiparous dams [30], severe metabolic acidosis may develop, which endangers the chances of survival of newborn calves [15,33]. In contrast, Vannouchi et al. [34] have recently reported that calves born after 2 h of calving showed decreased vitality, hypercapnia, hypoxia, and increased antioxidant status (glutathione peroxidase) due to respiratory acidosis while severe metabolic acidosis did not develop. All these results call attention to further studies to determine precisely the reasons that lead to a shift in the blood gases and acid-base parameters during calving. Therefore, it was hypothesized that by measuring the metabolic parameters like BE, HCO_3_^−^, and TCO_2_ besides pH and blood gases, respiratory and/or metabolic acidosis in the fetus to be born could be accurately differentiated.

The present study aimed to determine the changes in maternal and fetal arterial acid-base variables during the last days before and during calving in chronically catheterized Dutch-Friesian dams and their fetuses.

## 2. Materials and Methods

### 2.1. Animals and Surgery

The Veterinary Faculty Council (the State University of Utrecht, The Netherlands) approved the use and treatment of animals in this study. After buying healthy pregnant animals, fetal and maternal catheterization of nine pluriparous Dutch-Friesian pregnant cows (body condition score between 3.2 to 3.5) was performed at the Department of Veterinary Obstetrics, Gynaecology, and A.I. (Utrecht) as previously described by Taverne et al. [27]. Briefly, after withholding food for 48 h and water for 24 h operation was performed under general anesthesia in dorsal recumbency. The animals were premedicated intravenously with acepromazine (10 mg/100 kg bodyweight: Vetranquil R, Clin-Midy, France) and atropine (2 mg/100 kg bodyweight). Anesthesia was induced by intravenous infusion of a mixture of guaifenesin (10 g/100 kg bodyweight; Gujatal R, Aesculaap, The Netherlands) and thiopental sodium (0.7 g/100 kg bodyweight; Nesdonal R, Rhone Poulenc, France). The cow was intubated and ventilated with 2–3% fluothane in oxygen/nitrous oxide.

Following surgical preparation, the uterus was approached through a ventral midline incision. The right or left fetal hind limb was identified by intraabdominal palpation and moved so that the foot lay in the abdominal incision and under an intercotyledonary area of the uterus. The fetal leg was withdrawn from the uterus until the anterior surface of the hock joint was easily accessible. The fetal anterior tibial artery was catheterized with a polyvinyl catheter (0.75 mm I.D. × 1.45 mm O.D., Dural Plastics, Minto, Australia) which was advanced approximately 50 cm to lie with its tip in the dorsal aorta. The uterus was closed with one row of continuous sutures, which included all fetal membranes (chorioallantoic and amniotic membranes) and the uterine wall. The second row of continuous Lembert sutures was used to cover the first row. The catheter was exteriorized through the lateral abdominal wall and temporarily wrapped in a sterile towel. The abdominal midline incision was then closed using standard procedures.

Subsequently, the cow was positioned on her right side and prepared for cannulation of the left circumflex iliac artery. A polyvinyl catheter (1.02 mm I.D. × 1.78 mm O.D., Norton Plastics, Hayward, CA, USA) was advanced approximately 40 cm to lie with its tip in the dorsal aorta of the cow. Finally, both catheters were tunneled subcutaneously to the most dorsal area of the sub-lumbar fossa. They were fixed to the skin and protected within gauze swabs soaked in alcohol within a plastic bag. A more detailed description of this operation illustrated with several pictures has been published recently [28]. On the morning following surgery, the cow was placed in a pen, where she remained until after calving. The outer ends of the catheters were transferred to a hood containing a small container of alcohol. Hypodermic needles capped with Luer-lock injection caps were inserted into the ends of the catheters, which were filled with heparinized saline (200 IU/mL) between samplings.

The mean (±SD) gestational age on the day of surgery was 266 ± 13 (between 254 and 279) days (Table 1) and the cows had their second to fourth gestation (3.2 ± 0.7).

### 2.2. Evaluation of Neonatal Vitality

Neonatal vitality was evaluated immediately after delivery using the vitality score recommended by Szenci [35]. The following categories were used: a score of 3 indicated normal tonicity, head erect, and regular reflectory movements; a score of 2 indicated low tonicity, sternal recumbency with fetal head requiring support, reduced number and intensity of reflectory movements; and a score of 1 indicated toneless, head dropping, limbs extended, and cardiac activity present and a score of 0 indicated toneless, head dropping, limbs extended, and cardiac activity absent (dead).

### 2.3. Blood Sampling Protocol

Arterial blood samples were taken daily, usually between 8.00 and 12.00 a.m., starting on the sixth day (between 4 and 9 days) after surgery. When cows showed signs of impending parturition, sampling was repeated. Immediately after calving, blood samples were withdrawn from the newborn calves. On several occasions, samples could not be obtained (blocked catheters, unobserved start of calving), therefore, a strict protocol could not be followed in each of the cows. In two cows, the clinical signs of impending parturition were not expressed, therefore, we could withdraw the last blood samples only 17 and 23 h before calving, respectively. As the acid-base parameters did not differ from the other animals they were not removed from the evaluation.

Before arterial blood sampling, heparinized saline solution was removed from each catheter, and 1 mL of blood was withdrawn for acid-base measurements as described previously [36]. Briefly, the dead space of a 2-mL plastic syringe and an attached needle was filled with heparin solution (1000 IU/mL), and blood samples were taken anaerobically. Air bubbles observed in the sample were removed immediately, and the sample was mixed by rolling. The syringe was capped with a rubber stopper and placed on a bed of crushed ice. In each case, arterial blood samples were analyzed for acid-base variables (pH, pCO_2_, pO_2_, SaO_2_, BE, HCO_3_^−^ and TCO_2_ using an acid-base analyzer (AVL Gas-Check 938, AVL Medical Instruments, Graz, Austria) at 37 °C within 15 min. After sampling, heparinized saline solution was used again to remove the blood from the catheter. After closing the outer end of the catheter with a Luer-lock injection cap, it was put back into a small container filled with alcohol. After calving, the fetal catheter was cut. A hypodermic needle capped with a Luer-lock injection cap was inserted into the end of the catheter to continue the withdrawal of additional arterial blood samples.

### 2.4. Statistical Analysis

Data were analyzed using R3.5.2. Statistical software [37]. Acid-base data were checked for normality with the Shapiro–Wilk test. In prepartum samples, the mean, the standard deviation (SD), and the 5th and 95th percentiles were calculated for both the dams and the fetuses. We used a generalized linear model to determine the effect of sample number for each acid-base variable, using sample number and animal ID as factors. Pearson’s product-moment correlation was used to determine the prepartum correlation between fetal and maternal acid-base values. Each parameter from both maternal and fetal samples taken around the onset of calving was compared to the prepartum and the immediate prepartum samples in the dam and the immediate fetal prepartum and the immediate neonatal post-partum values using two-sided paired *t*-tests. In the case of newborn calves, according to the BE values (0.7 to −1.7 mmol/L vs. −3.7 to −7.2 mmol/L), two neonatal groups were established and analyzed separately. A probability of *p* < 0.05 was considered statistically significant.

## 3. Results

All cows recovered well after surgery, and the mean interval between operation and calving was 19 ± 6 (SD) days, resulting in mean gestational age of 285 ± 8 (between 273 and 300) days at calving. We were able to take an average of 11 ± 4 (between 6 to 17) blood samples from the cows and 12 ± 4 (between 7 to 17) blood samples from eight fetuses because one catheter was blocked before calving (Table 1). All single calves without obstetrical assistance were born alive within one h after the hooves appeared in the vagina in anterior presentations, and each newborn calves had a normal vitality score (Score 3). Neonatal blood samplings were continued after birth through the same catheter. After calving, 4 of 9 cows had retained fetal membranes.

The mean (SD) and 5th and 95th percentiles of the maternal blood gases and acid-base parameters before calving (*n* = 100) and about ≤2 h before calving (*n* = 9) are given in Table 2. The prepartum maternal acid-base values showed no significant differences over time until about ≤2 h before calving, although some individual maternal differences were observed between animals. Regarding the maternal arterial acid-base variables, only the pH values increased significantly (*p* < 0.001). Conversely, pCO_2_ and pO_2_ values decreased significantly (*p* < 0.01 and *p* = 0.01, respectively) until about ≤2 h before calving, demonstrating the effect of imminent calving on pH and blood gases. At the same time, maternal prepartum acid-base values did not show any differences between cows with and without retained fetal membranes after calving.

The mean (SD) and 5th and 95th percentiles of the fetal/neonatal blood gases and acid-base parameters before calving (*n* = 96) and about ≤2 h before calving (*n* = 8) as well as immediately after calving (*n* = 9) are given in Table 3. We could withdraw the arterial blood sample from the missing calf after birth, and it showed similar acid-base values (BE: 0.0 mmol/L) to those for the three newborn calves; therefore, these values were not removed. The prepartum fetal acid-base values showed no significant differences over time until about ≤2 h before calving, although some individual differences were observed between fetuses. At the same time, the neonatal pH and BE values (*n* = 9) were significantly lower (*p* < 0.01 and *p* = 0.03, respectively), while pCO_2_ was significantly higher (*p* = 0.03) in the immediate post-partum samples compared to prepartum samples (data not shown).

Except for SaO_2_, all acid-base parameters between maternal and fetal arterial blood showed a significant positive correlation before calving. In contrast, from about ≤2 h before calving, only pCO_2_ and acid-base parameters (BE, HCO_3_^−^ and TCO_2_) showed a significant positive correlation albeit to a reduced extent (Table 4).

Among the nine newborn calves, there were four newborn calves with similar BE values (0.7 to −1.7 mmol/L) immediately after birth than those before delivery. Only pCO_2_ showed a significant increase (*p* = 0.02) compared to samples taken 23 ± 13 min (except for one case when it was 23 h) before calving (Table 3). Concurrently, post-partum pH tended to be lower (*p* = 0.08). In these four calves, after birth, physiological respiratory acidosis dominated. In the remaining five newborn calves, the mean pH and BE values were significantly lower (*p* < 0.01) after birth, while pCO_2_ tended to be higher (*p* = 0.06) than in samples taken 124 ± 43 min (except for one case when it was 17 h) before calving. Three newborn calves were born with physiological respiratory and metabolic acidosis, while the remaining two had moderate respiratory and metabolic acidosis (pH: 7.167 and 7.089, while BE: −6.6 and −7.2 mmol/L, respectively). None of the newborn calves were lost in the post-parturient period.

## 4. Discussion

In agreement with previous findings [22,29,30], catheterized dams and fetuses’ arterial blood pH and blood gases are very stable during late gestation. Our results also apply to the other acid-base variables like BE, HCO_3_^−^, and TCO_2_ (Table 2 and Table 3). The daily maternal and fetal blood pH and blood gases also changed a little up to the day of birth. Differences between maternal and fetal blood gases ensured the placental transfer of blood gases, while there was a significant pH gradient between the fetal and maternal arterial blood. According to Comline and Silver [22], the differences between maternal and fetal arterial samples were 0.052 for pH, 7.8 mmHg (1.04 kPa) for pCO_2_, and 60.8 mmHg (8.1 kPa) for pO_2_, respectively. In our case (*n* = 86, data not shown), the mean differences calculated from the blood withdrawn from the aorta were very similar: 0.075 for pH, 1.46 kPa for pCO_2_, and 9.92 kPa for pO_2_, respectively. Sangild et al. [29] also reported similar pH value differences (0.080 vs. 0.074, respectively) for dams with in-vitro produced (IVP) embryos and dams with AI embryos between 2–5 days after surgery during late pregnancy. In comparison, there were somewhat higher differences regarding the maternal and fetal pCO_2_ values (IVP dam vs. fetus: 11.9 mmHg /1.58 kPa/ and AI dam vs. fetus: 26.1 mmHg /3.48 kPa/) between 2 to 5 days after surgery. According to our results, the other acid-base parameters (BE, HCO_3_^−^ and TCO_2_) were hardly different.

Except for SaO_2_, all acid-base parameters between maternal and fetal arterial blood showed a significant positive correlation before calving. In contrast, from about ≤2 h before calving, only pCO_2_ and acid-base parameters (BE, HCO_3_^−^ and TCO_2_) showed a significant positive correlation, albeit to a reduced extent, indicating the changes caused by uterine contraction at the beginning of calving (Table 4).

It was also found by Comline et al. [20] that fetal blood pH remained stable both immediately before and during normal parturition, and it was only after rupture of the umbilical cord that there was a fall in blood pH associated with a high pCO_2_ level. In contrast, Wilson et al. [30] indicated that fetal pCO_2_ values might elevate in the last 24 h before birth. According to our results, we had four newborn calves that showed little difference between prenatal and postnatal pH values (−0.035). In contrast, pCO_2_ values started to increase significantly (*p* = 0.02), indicating a shift towards physiological respiratory acidosis. In contrast, the pO_2_ and SaO_2_ showed some non-significant improvements (1.28 kPa, 20.2 %) immediately after birth. At the same time, the other acid-base parameters did not differ. The remaining five newborn calves showed a significant decrease in arterial blood pH (*p* < 0.01) and BE (*p* = 0.01), while pCO_2_ tended to be higher (*p* = 0.06), indicating a shift towards physiological respiratory and metabolic acidosis. At the same time, the other acid-base parameters hardly differed. It is essential to mention that metabolic acidosis developed gradually in four newborn calves during about ≤2 h of calving, while in the remaining newborn calf during the last 3 min of birth because immediately before birth, the BE value was 0.4 mmol/L and immediately after birth it was −5.4 mmol/L. Metabolic acidosis appears to be due to electrolyte imbalance and L-lactate accumulation due to anaerobic glycolysis for no tissue oxygenation.

In the field, the prevalence of normal fetuses (physiological acidosis: pH > 7.2) before spontaneous delivery and obstetrical assistance withdrawing venous blood from *v. metacarpalis volaris superficialis* [38,39] or capillary blood [40] can vary between 74.2 and 85% [38,39,40], while before Caesarean section withdrawing blood from *v. umbilicalis* or *v. digitalis dorsalis communis III* [14,41,42] before extraction from the uterus between 51.2 and 63.6%, respectively. A close correlation could be found between the duration of the second stage of calving and the duration of obstetrical assistance because the prevalence of severely acidotic calves (pH < 7.0) can be increased if the duration of the second stage of calving is extended (<2 h: 0%, 2 to 4 h: 19%, 4 to 7 h: 44%: [15]). In contrast, besides respiratory acidosis, Vannouchi et al. [34] reported normal mean metabolic parameters (BE: −5.4 mmol/L, HCO_3_^−^: 20.5 mmol/L, TCO_2_: 21.9 mmol/L) even if the duration of calving was longer than >4 h. At the same time, a significant respiratory depression (altered lung gas exchange and delayed lung clearance) was also observed [43]. Therefore, it is important to emphasize for the dairy practice that, in agreement with Comline and Siver [22], not only the pH value and the blood gases but the other acid-base variables will start to move in the direction of expressed respiratory and metabolic acidosis only after appearing the amniotic sac and fetal feet in the vulva during the second stage of labor. Therefore, it is crucial to start obstetrical assistance within 70 min after amniotic sac appearance or 65 min after the appearance of feet in the vulva [44]. If we start obstetrical assistance too early, we may increase not only the prevalence of stillbirth rate but the prevalence of injuries to the birth canal and retained fetal membranes [45]. At the same time, Villettaz Robichaud et al. [46] could not confirm the negative effect of early intervention (within 15 min after the first sight of both front hooves in the vulva) on the stillbirth rate; however, sterile obstetrical lubricant was applied liberally to the dam’s birth canal around the fetus before performing the examination and providing obstetrical assistance. The effect on the prevalence of injuries of the birth canal and retained fetal membranes was not reported. At the same time, Vannouchi et al. [47] could not confirm any connection between retained fetal membranes and extended duration of calving (>4 h). In our case, the prepartum maternal acid-base parameters did not differ if retained fetal membranes occurred after calving. In contrast, dystocia [15,16,35,40] or extended duration of obstetrical traction (more than 2 min) [33] may also contribute to the development of expressed respiratory and metabolic acidosis in the fetus being born.

## 5. Conclusions

To decrease the prevalence of dystocia and stillbirth and to improve animal welfare, one of our most important management activities during the periparturient period is to provide obstetrical assistance at an appropriate time after detecting the onset of the second stage of labor. In addition, maternal and fetal cannulation can help us understand those physiological and pathological changes that may endanger the fetuses’ life to be born. This is all the more important because apart from buffering the metabolic acidosis in a dairy farm, instruments for effectively clearing the airways from amniotic mucus and providing artificial respiration are still not widely available; therefore, we must emphasize the importance of prevention of neonatal asphyxia.

## Figures and Tables

**Table 1:** The data of catheterized cows and fetuses.

Cow	Duration of Gestation (Days)	Duration between Operation and Calving (Days)	Period of Sampling (Post-Surgery)	Number of Maternal Blood Samples before Calving	Number of Fetal Blood Samples before Calving	Retained Fetal Membranes (Y/N)
A	254	31	D9–D31	10	16	Y
B	269	25	D6–D25	14	12	N
C	262	11	D6–D11	6	8	Y
D	258	16	D6–D16	10	11	N
E	265	14	D4–D14	11	*	Y
F	261	17	D5–D17	7	7	Y
G	NK	12	D5–D12	8	8	N
I	277	20	D5–D20	17	17	N
J	279	21	D5–D21	17	17	N
Mean (±SD)	266 ± 9	19 ± 6	6 ± 1 **	11 ± 4 N = 100	12 ± 4 N = 96	4/5

NK: not known, * fetal catheter was blocked, ** First blood samples were withdrawn after operation.

**Table 2:** Changes in maternal arterial blood gases and acid-base parameters in catheterized cows (n = 9) before calving and about ≤2-h before calving. Due to daily sampling from dams, 100 arterial blood samples could be withdrawn.

Acid-Base Parameters	Maternal Arterial Blood			Maternal Arterial Blood about ≤2-h before Calving	*p*-Value
	Mean ± SD	5th Percentile	95th Percentile	Mean ± SD	
pH	7.427 ± 0.029	7.381	7.467	7.447 ± 0.029	0.001
pCO_2_ (kPa)	5.02 ± 0.41	4.30	5.60	4.68 ± 0.31	0.01
pO_2_ (kPa)	13.43 ± 1.63	11.30	16.87	12.64 ± 1.77	0.01
SaO_2_ (%)	96.9 ± 0.8	95.6	98.1	96.7 ± 1.1	0.09
BE (mmol/L)	0.56 ± 1.92	−2.72	3.72	1.3 ± 2.1	0.22
HCO_3_^-^ (mmol/L)	24.5 ± 2.0	20.6	27.4	24.6 ± 2.0	0.82
TCO_2_ (mmol/L)	25.6 ± 1.9	22.3	28.4	25.1 ± 1.5	0.58

SD: standard deviation.

**Table 3:** Changes in fetal arterial blood gases and acid-base parameters before calving and about <2-h before calving in catheterized fetuses and immediately after calving in newborn calves.

Acid-Base Parameters	Fetal Arterial Blood			Fetal Arterial Blood about ≤2-h before Birth (n = 8 *)	Neonatal Arterial Blood *(Group A: n = 4)*	Neonatal Arterial Blood *(Group B: n = 5)*	*p*-Value	
	Mean ± SD	5th Percentile	95th Percentile	Mean ± SD	(Mean ± SD)	(Mean ± SD)	Fetal ≤2-h vs. Neonatal (*Group A*)	Fetal ≤2-h vs. Neonatal (*Group B*)
pH	7.351 ± 0.019	7.326	7.384	7.347 ± 0.019	7.312 ± 0.019	7.209 ± 0.073	0.08	0.01
pCO_2_ (kPa)	6.52 ± 0.48	5.89	7.26	6.35 ± 0.70	7.24 ± 0.26	8.26 ± 1.81	0.02	0.06
pO_2_ (kPa)	3.61 ± 0.65	2.43	4.45	2.83 ± 0.66	4.11 ± 0.86	3.19 ± 0.61	0.49	0.16
SaO_2_ (%)	47.9 ± 11.9	23.2	62.6	31.7 ± 13.2	51.9 ± 14.7	30.9 ± 11.0	0.63	0.64
BE (mmol/L)	0.51 ± 1.28	−1.66	2.72	−0.4 ± 2.0	−0.3 ± 0.9	−5.4 ± 1.3	0.43	0.01
HCO_3_^-^ (mmol/L)	26.5 ± 1.7	24.1	29.8	25.6 ± 2.6	26.8 ± 0.7	23.4 ± 1.8	0.77	0.43
TCO_2_ (mmol/L)	27.6 ± 1.4	25.4	29.6	26.2 ± 2.0	28.4 ± 0.7	24.5 ± 1.5	0.63	0.83

* Due to the blocking of a fetal catheter, blood sample could not be withdrawn before calving.

**Table 4:** Correlation between maternal and fetal acid-base parameters before calving.

Acid-Base Parameters	Before Caving			About ≤2-h before Calving		
	Pearson’s Correlation	Confidence Intervals	*p*-Value	Pearson’s Correlation	Confidence Intervals	*p*-Value
pH	0.47	0.29 to 0.62	0.001	0.57	−0.22 to 0.91	0.14
pCO_2_ (kPa)	0.48	0.30 to 0.63	0.001	0.87	0.42 to 0.98	0.01
pO_2_ (kPa)	0.28	0.07 to 0.46	0.01	0.44	−0.38 to 0.87	0.27
SaO_2_ (%)	−0.01	−0.23 to 0.21	0.93	0.28	−0.53 to 0.82	0.51
BE (mmol/L)	0.44	0.26 to 0.6	0.001	0.81	0.24 to 0.96	0.02
HCO_3_^-^ (mmol/L)	0.43	0.24 to 0.59	0.001	0.77	0.03 to 0.96	0.04
TCO_2_ (mmol/L)	0.50	0.30 to 0.65	0.001	0.88	0.47 to 0.98	0.01

## Data Availability

The data presented in this study are available on request from the corresponding author.
